# A statistically established reference value determined for the Vaxarray Coronavirus (CoV) seroassay to characterize vaccination and natural infection

**DOI:** 10.1186/s12879-024-10117-5

**Published:** 2024-11-15

**Authors:** Francisco Mimica Porras, Gabriel Pineda, Abigail Mangilog, Keith Hernandez, Cynthia Sikorski, Michelle Lane

**Affiliations:** 1 US Naval Health Research Center, Operational Infectious Diseases, San Diego, CA USA; 2 General Dynamics Information Technology, Falls Church, VA USA

**Keywords:** SARS-CoV-2, Vaxarray Coronavirus (CoV) seroassay, Reference value, Cutoff, Student *t*- distribution, Nucleocapsid protein, Surrogate virus neutralization assay (sVNT)

## Abstract

Serological diagnostic tests are available that measure antibody levels against SARS-CoV-2 antigens. We utilized the Vaxarray Coronavirus (CoV) seroassay, which measures SARS-CoV-2 IgG antibodies against the full-length spike protein (FLS), receptor binding domain (RBD), and S2 extracellular domain (ECD). Previous serological studies have used reference values that have not been validated and require many samples. Here, we show statistically established reference values determined using the upper tail of the Student *t*-distribution method. The target population was any personnel age 18 years and older working on a U.S. Navy ship, and vaccinated with Wuhan variant. The relative fluorescence mean (RFM) reference values for the full-length spike protein, RBD, and S2 ECD were 17,731, 13,990 and 9096, respectively. By using generalized non-parametric regression and reference values for the RBD spike protein and S2 ECD of SARS-CoV-2, this study was able to distinguish vaccine-mediated immune responses from natural infections. We provide the method and statistical code as a resource to determine future reference values for other serological assays.

**Supplementary Information:**

The online version contains supplementary material available at 10.1186/s12879-024-10117-5.

## Introduction

During the SARS-CoV-2 pandemic, the U.S. healthcare system was challenged with high volumes of patients who required critical care. To preserve and efficiently utilize healthcare resources, it became imperative to properly stratify patients for care [5, 13]. To accomplish this goal, it has become important to develop diagnostics for SARS-CoV-2. Nucleic acid diagnostics (i.e., quantitative polymerase chain reaction (qPCR)) focus on the identification of infections; these assays have high sensitivity and specificity in identifying SARS-CoV-2 [14]. However, they are incapable of determining an individual’s immune response to infection or vaccination [9].

The determination of an individual’s exposure and infection status can be measured by an individual’s immune response to SARS-CoV-2. This information is critical for establishing the seroprevalence and vaccination response [2]. The extent of the immune response impacts seroprotection, recovery time, and antibody longevity [4].

In addition, vaccine development is a critical response for controlling the spread of a pandemic to a novel infectious agent for which the population has limited immunity. A better understanding of the host’s response to vaccines and natural infection is necessary, as viral agents that replicate quickly have greater chances of producing variant strains, making it more difficult to control the spread of the infection.

A variety of serological diagnostic methods can be used for SARS-CoV-2 testing. Currently, there are more than 300 serological tests that have received emergency use authorization (EUA) from the United States Food and Drug Administration (FDA) on the market. The field of serological diagnostics is composed of a variety of methods and standards [11].

In this study, three commercial assays were used to attempt to delineate the host response to vaccination from natural infection. The first assay Coronavirus (CoV) seroassay utilized the Vaxarray platform (InDevR, Inc., Boulder, CO). Previous studies using this assay have reported the linear dynamic range, limit of detection, specificity, reproducibility, and accuracy [6]. Other studies utilizing this microscale, multiantigen array platform have characterized and validated influenza vaccine antigens [8]. The CoV seroassay measured the relative fluorescence of IgG antibodies against nine different antigens including 3 SARS-CoV-2 antigens. The assay setup is comparable to microarray testing platforms, as each protein (antigen) is spotted in replicate on a glass slide that can test 16 samples simultaneously. The following nine antigens are used in the CoV seroassay: full-length spike protein(FLS), receptor binding domain protein (RBD), S2 extracellular domain (S2 ECD) of SARS-CoV-2, and spike protein from SARS, MERS, HKU1, OC43, NL63, and 229E [1]. The SARS-CoV-2 recombinant antigens utilized in CoV seroassay are produced from Wuhan variant sequence.

The second serological assay used is a competition-based ELISA that functions as a surrogate of virus neutralization [12]. The assay is called the sVNT assay (GenScript, Piscataway, NJ) which measures antibodies that inhibit the interaction between the ACE2 receptor and the receptor binding domain of the SARS-CoV-2 spike protein.

The third assay used was the Platelia SARS-CoV-2 total nucleocapsid assay. Results from this assay distinguish antibodies generated by vaccine or natural infection since the coding sequence for the nucleocapsid antigen is not included in the vaccine. Individuals who displayed a response against nucleocapsid would represent individuals who have been previously infected with SARS-CoV-2. The nucleocapsid assay was used to identify individuals that were not infected with SARS-CoV-2.

Serology testing results are typically presented as a limited dilution series or two to three standard deviations from the mean negative control reading [7]. Here, we present an established reference value for the full-length spike protein, RBD, and S2 ECD of the SARS-CoV-2 antigen using the upper tail of the Student *t*- distribution method [3]. In addition, we provide the method and statistical code as a resource to determine future reference values for other serological assays.

## Methods

### Study design and ethical considerations

The study design was cross-sectional and was approved by the Institutional Review Board (IRB) of the Naval Health Research Center (NHRC). The IRB protocol used was NHRC.2021.0009.

### Serum sample collection and processing

One hundred and six vaccinated US. Navy active-duty personnel enrolled in our study. Enrollment was voluntary and informed consent to participate was obtained from all participants in the study. The target population was any personnel aged 18 years and older working on a Navy ship. Nasal swab and venous blood samples were collected for SARS-CoV-2 PCR and serologic testing, respectively. Serum samples were collected from individuals utilizing BD Vacutainer Serum Separator tubes (BD 3680). Serum was processed by centrifuging the samples in separator tubes for 15 min at 3000 RPM, and the separated serum was stored at -80°C in cryovials. The samples underwent two freeze thaw cycles to complete testing for the study.

### Multiplex immunoassay detection of anti − SARS-CoV-2 antigen-specific IgG

The VaxArray Coronavirus (CoV) SeroAssay (cat# VXCV-5100, InDevR, Inc., Boulder, CO) kit utilizes nine different recombinant protein antigens that are spotted onto a glass slide and compose an array. Each array detects and measures antigen–antibody (IgG) interactions. The CoV seroassay was performed according to the manufacturer’s instructions for use and has been described previously [1]**.** All SARS-CoV-2 antigens are based on Wuhan variant. In brief, antigen is the full-length SARS-CoV-2 spike protein, which contains both the S1 and S2 domains (amino acids: 1 – 1273). The second antigen is the RBD (amino acids: 319 – 541) of the SARS-CoV-2 spike protein. The third antigen is the S2 extracellular domain (ECD) (amino acids: 686 – 1213) of the SARS-CoV-2 spike protein. The fourth antigen, SARS, is the S1 domain of the SARS spike protein. The fifth antigen, MERS, is the S1 domain of the MERS spike protein. The sixth antigen, HKU, is the S1 domain of the HKU spike protein. The seventh antigen, OC43, is the full-length OC43 spike protein. The eighth antigen, 229E, is the S1 domain of the 229E spike protein. The ninth and final antigen, NL63, is the S1 domain of the NL63 spike protein. All proteins were expressed in mammalian cells except for antigens 3 and 7, which are expressed in insect cells. Before use, all the reagents and glass slides were moved to 20°C, room temperature for at least 30 min. The specimens were diluted in protein blocking buffer 2.0 (cat# VX-6305) 1:100 and 1:200, including the standards, and^1^ incubated for 60 min at 20°C, room temperature in a humidity chamber prepared as described in the manufacture’s operation manual. Following incubation, the samples were removed, and the slides were washed with 50 µl of wash buffer 1 (cat# VX-6303). The labeled anti-human IgG (cat# VXCV-7623) was diluted 1:10 in protein blocking buffer, and 50 µl was added to the slide. Following 30 min of incubation at 20°C, room temperature in the humidity chamber, the label was removed, and the slides were sequentially washed once with the following solutions: wash buffer 1, wash buffer 2, 70% ethanol, and purified water. Following all washes, the slides were dried using the VaxArray slide drying station (cat# VX-6208, InDevR, Inc.) and imaged using the VaxArray Imaging system.

### Detection of Anti-SARS-CoV-2 Neutralization Antibodies using Surrogate Virus Neutralization Assay

A SARS-CoV-2 surrogate virus neutralization assay kit (cat# L00847A, GenScript, Piscataway, NJ) was used to measure neutralizing antibodies. Before use, all required reagents and assay plates were moved to room temperature for at least 30 min. The assay was run according to the manufacturer’s instructions (IFU). Serum was incubated with horseradish peroxidase (HRP)-conjugated RBD at 37°C for 30 min, and then the mixtures were placed in 96-well plates precoated with human angiotensin-converting enzyme 2 (hACE2) proteins and incubated at 37°C for 15 min. After the wells were washed, 3,3',5,5'-tetramethylbenzidine (TMB) and stop solution were added to each well. Finally, the optical density of each well was read at 450 nm and 620 nm. The presence of RBD/ACE2 blocking antibodies in an individual specimen was determined by the absorbance (450/620 nm) measured with a DYNEX Agility (Chantilly, VA).

### Detection of Anti − SARS-CoV-2 Nucleocapsid Antigen-Specific antibodies by ELISA

A Platelia SARS-CoV-2 Total Ab ELISA kit (cat# 12,015,253, Bio-Rad, Hercules, CA) was used to measure and detect total anti-SARS-CoV-2 nucleocapsid antibodies (IgM/IgG/IgA) in human serum. Before use, all required reagents and assay plates were moved to room temperature for at least 30 min. A 1:5 dilution (15 µl of serum:60 µl of dilution buffer) of each serum sample was added to a predilution microplate well and mixed with 75 µl of conjugate recombinant SARS-CoV-2 nucleocapsid protein coupled with horseradish peroxidase. Immediately, 100 μL of the prediluted controls and serum samples were added to the wells of the reaction microplate. The reaction plate wells were coated with the recombinant SARS-CoV-2 nucleocapsid protein. The reaction plate was then sealed with an adhesive plate seal to minimize evaporation and incubated at 37°C for 60 min. At the end of the incubation period, the reaction plate was washed 5 times using a DYNEX DS2® (Chantilly, VA) microplate washer with 800 μL of wash solution per well. After the wash, the microplate was inverted and gently tapped on absorbent paper to remove the remaining liquid. Once complete, 200 μL of the development solution (TMB substrate buffer-R8 and Chromogen-R9) was quickly added to each well and incubated at room temperature for 30 min in the dark without an adhesive plate seal. Following incubation, 100 μL of stop solution was added to each well and mixed thoroughly using the same sequence and rate of addition as for the development solution. Finally, the optical density of each well was read at 450 nm and 620 nm.

### Statistical computations utilizing R code were performed for reference value determination for the FLS and RBD antigens on the CoV Seroassay

The Student *t*-distribution equation shown below allows the user to modify the confidence level of the reference value. The following method began by testing 106 samples on the Coronavirus (CoV) SeroAssay generating relative fluorescent mean (RFM). To determine the background signal on the CoV Seroassay for the FLS and RBD antigens, 106 individuals were tested using the surrogate virus neutralization assay (sVNT). Individuals that had a negative result did not generate an immune response and were used to determine the background RFM for the full spike protein and RBD on the CoV Seroassay. Background measurements on the CoV Seroassay are required to determine reference values as they serve as negative controls in the upper Student *t-*distribution calculation for FLS and RBD.

### Statistical computations utilizing R code were performed for reference value determination for the S2 ECD antigen on the CoV Seroassay

Reference value determination for the S2 ECD began by testing 106 samples on the Coronavirus (CoV) seroassay producing relative fluorescent mean (RFM). To determine the background signal on the CoV seroassay for the S2 ECD antigen, 106 individuals were tested using the Platelia SARS-CoV-2 nucleocapsid antibody assay. A negative result identified individuals who have not been infected by SARS-CoV-2. These negative individuals establish the background RFM for the S2 ECD antigen on the CoV Seroassay. Background measurements on the CoV seroassay are required to determine reference values as they serve as negative controls in the upper Student *t-*distribution calculation for S2 ECD.

The method is sensitive to outlier readings. To mitigate the inclusion of outliers in our analysis, the selection of negative controls was random and included a minimum of nine negative controls and a maximum of twenty negative controls [3]. The relative fluorescence mean (RFM) readings of the outliers were below the mean plus one standard deviation from the pool of negative samples by the CoV seroassay, respectively. In the equation below, x̄ is the mean of negative control readings.

$$ReferenceValue=\text{ }\overline{\mathrm x}\pm\text{SD }t\sqrt{1+\left(\frac1{\text{n}}\right)}$$

SD is the standard deviation, n is the number of negative controls, and t is the (1-α) percentile of the one-tailed Student *t*-distribution with v = n – 1 degrees of freedom. The R code for calculating the upper Student *t*-distribution method is included in (Supplemental material 1). To utilize the R code you will need to have R and Excel installed on your computer. To complete the calculations, you need to copy and paste the R code into the R program. Download the excel files we have included in manuscript as a template for your own data use and save file on your computer desktop. Replacing our RFM data with your own data and again saving the file on your desktop. In the R code you should revise the section labeled as “Write Computer Username” with your own computer’s username. Then copy it into the R Script file and save it and run the code.

## Results

All 106 subjects in this study were vaccinated against the SARS-CoV-2 Wuhan variant, which expresses the full-length SARS-CoV-2 spike protein. Subjects were also PCR negative for SARS-CoV-2 at the time of enrollment. We began to statistically determine the cutoff values for all three antigens (FLS, RBD, S2 ECD) for the Coronavirus (CoV) seroassay. Relative fluorescent mean (RFM) data for all three antigens is shown in Table 1. The FLS mean for the 106 individuals tested was 43,900 ± 21,800 RFM (mean ± SD). The range of the FLS signal was 1770 – 64,400 RFM (min, max). The RBD mean for the 106 individuals tested was 38,800 ± 23,000 RFM (mean ± SD). The range of the RBD RFM signal was 1580 – 64,400 RFM (min, max). The S2 ECD mean for the 106 individuals tested was 15,200 ± 18,500 (mean ± SD). The range of the S2 ECD RFM signal was 673 – 64,300 (min, max).

**Table 1:** Relative fluorescent mean (RFM) data from SARS-CoV-2 antigens using the CoV-2 seroassay from 106 individual serums tested

	Overall (*N* = 106)
**Full-length spike protein/InDevR**	
Mean (SD)	43,900 (21,800)
Median [Min, Max]	54,500 [1770, 64400]
**RBD protein/InDevR**	
Mean (SD)	38,800 (23,000)
Median [Min, Max]	38,600 [1580, 64400]
**S2 extracellular domain/InDevR**	
Mean (SD)	15,200 (18,500)
Median [Min, Max]	4950, [673, 64300]

### Determining the reference values (cutoff) for the mammalian expressed full-length SARS-CoV-2 spike protein for the CoV seroassay

**Fig. 1:**
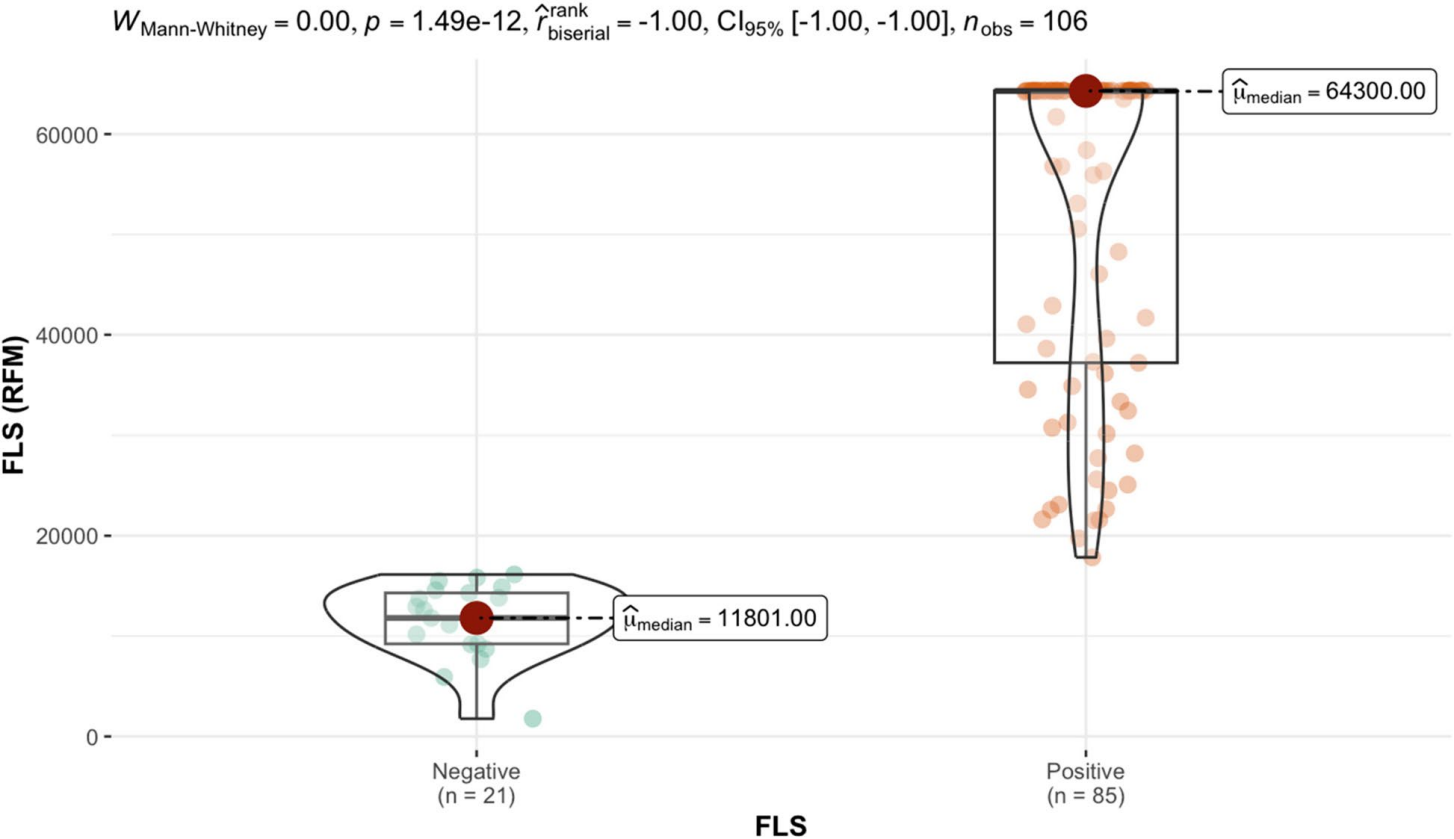
Mann–Whitney test of the distribution of relative fluorescent means (RFM) from full length spike antigen (FLS). Significant statistical difference (*p*-value < 0.000) between the positive (64,300) and negative (11,801) individuals based on reference value (cutoff) results

Serum from 106 individuals were tested on both the CoV seroassay and sVNT assay. Antibodies in each serum that bind to the full-length spike protein generate a relative fluorescent mean signal (RFM). The relative fluorescent mean (RFM) results for the full-length spike protein and antibodies measured in the sVNT assay are shown in Table 2. Out of the 106 individuals tested, 88.7% generated a positive response and 11.3% were considered a negative response based on sVNT results. Nine random individuals from the 106 tested with a negative result in the sVNT assay were considered as negative controls and listed in Table 2. We utilized the RFM values from the designated negative controls to calculate the upper tail of the Student *t*-distribution method for five distinct confident intervals (95.0%, 97.5%, 99.0%, 99.5%, 99.9%) for the full-length spike protein (Table 3). The 17,731 RFM at 95% confidence interval was determined as the reference value (cutoff). Relative fluorescent mean readings above 17,731 are classified as positive, while readings at or below the relative value are negative. Out of the 106 individuals tested, 80.2% generated a positive response and 19.8% were considered a negative response based on reference values cutoff results listed in Table 3. The median from positive FLS individuals is 64,300 RFM and the median for negative individuals is 11,801 RFM. Applying the Mann–Whitney test to the RFM data in Table 2 determined there is a significant statistical difference between (*p*-value < 0.000) the distribution of RFM medians between positive and negative individuals is depicted (Fig. 1.)

**Table 2:**
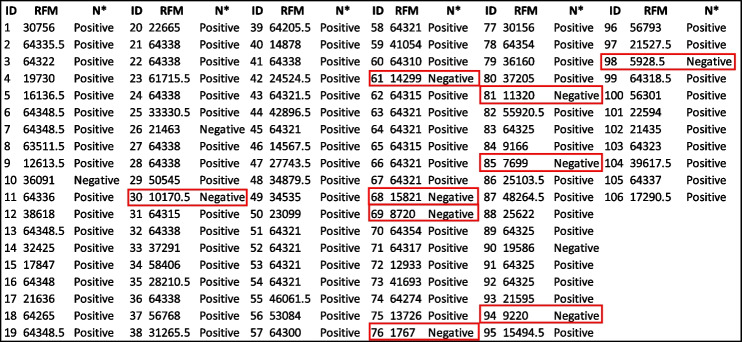
Full-length spike protein CoV2 Seroassay relative fluorescent mean (RFM) and Neutralization Antibodies from Surrogate Virus Neutralization Assay (sVNT) results from 106 individual serums

^*^Surrogate virus neutralization assay (sVNT)

negative controls

**Table 3:** The quantitative determination of the full-length spike protein reference value (cutoff)

Standard deviation multipliers (*f*) for calculation reference value					
**Number of Controls**	95.0%	97.5%	99.0%	99.5%	99.9%
2	26,602.29	49,646.22	118,541.80	233,292.20	1,151,160.00
3	15,399.21	20,261.64	29,622.04	40,031.69	83,643.44
4	14,096.28	16,938.60	21,594.88	26,052.16	41,045.52
5	13,707.13	15,835.87	19,040.83	21,871.45	30,355.54
6	13,894.15	15,725.66	18,344.86	20,544.60	26,681.09
7	14,443.58	16,157.47	18,524.88	20,446.41	25,550.68
8	16,135.08	17,994.49	20,499.86	22,483.81	27,570.18
9	17,731.31^a^	19,722.36	22,355.59	24,402.26	29,510.39

^a^Relative fluorescents mean cutoff for the full-length spike protein

### Determining the reference values (cutoff) for the mammalian expressed receptor binding domain (RBD) of SARS-CoV-2 spike protein for the CoV seroassay

**Fig. 2:**
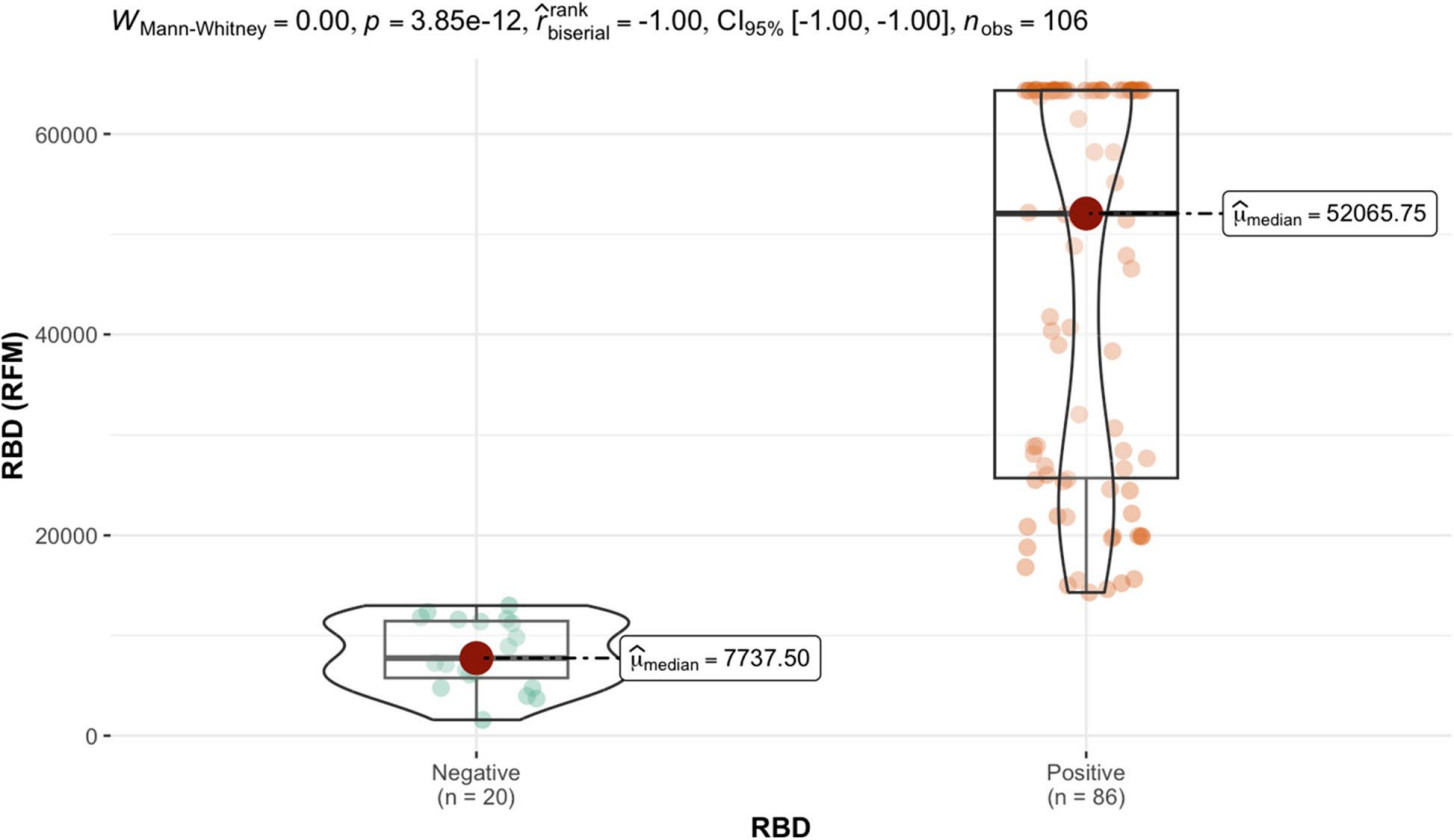
Mann–Whitney test of the distribution of relative fluorescent means (RFM) from receptor binding domain antigen (RBD). Significant statistical difference (p-value < 0.000) between the positive (52,065.75) and negative (7737.5) individuals based on reference value (cutoff) results

Antibodies against the receptor binding domain (RBD) were measure in serum from 106 individuals using the CoV seroassay. A fluorescent signal is generated from the antibody antigen interactions and is quantitated as the relative fluorescent mean (RFM). The relative fluorescent mean (RFM) results for the RBD protein and antibodies measured in the sVNT assay results are shown in Table 4. Out of the 106 individuals tested, 88.7% generated a positive response and 11.3% were considered a negative response based on sVNT results listed in Table 4. Ten random individuals with a negative response in the sVNT assay were considered negative controls. We utilized the designated negative control RFM values were used to calculate the upper tail of the Student *t*-distribution method for five distinct confident intervals (95.0%, 97.5%, 99.0%, 99.5%, 99.9%) for the receptor binding domain protein in Table 5. The 13,990 RFM at 95% confidence interval was determined as the reference value (cutoff). Out of the 106 individuals tested, 81.1% generated a positive response and 18.9% were considered a negative response based on reference values cutoff results listed in Table 5. Relative fluorescent mean readings above 13,990 are classified as positive, while readings at or below the relative value are negative. The median from positive RBD individuals is 52,065.75 RFM and the median for negative individuals is 7737.5 RFM. Applying the Mann–Whitney test to the RFM data in Table 4 determined there is a significant statistical difference between (p-value < 0.000) the distribution of RFM medians between positive and negative individuals is depicted (Fig. 2.)

**Table 4:**
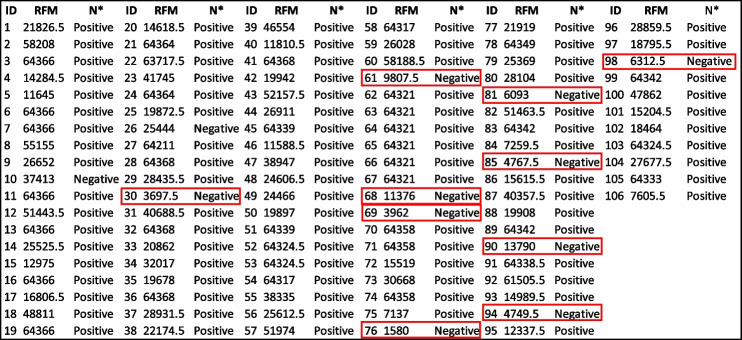
Receptor binding domain (RBD) Relative fluorescent mean (RFM) for the CoV2 Seroassay and Neutralization Antibodies from Surrogate Virus Neutralization Assay (sVNT) results from 106 individual serums

^*^Neutralizing antibody assay results

negative controls

**Table 5:** The quantitative determination of the receptor binding domain protein reference value (cutoff)

Standard deviation multipliers (*f*) for calculation reference value					
**Number of Controls**	95.0%	97.5%	99.0%	99.5%	99.9%
2	14,216.96	25,939.50	60,991.49	119,373.00	586,356.30
3	7481.97	9566.46	13,579.51	18,042.40	36,739.93
4	7059.85	8314.95	10,371.12	12,339.42	18,960.36
5	6794.15	7714.20	9099.43	10,322.86	13,989.79
6	7422.33	8326.81	9620.11	10,706.42	13,736.82
7	7779.81	8642.55	9834.18	10,801.45	13,370.84
8	9954.69	11,153.89	12,769.65	14,049.17	17,329.56
9	11,828.81	13,272.38	15,181.56	16,665.45	20,368.99
10	13,990.38^a^	15,716.95	17,967.61	19,691.57	23,904.80

^a^Relative fluorescents mean cutoff for the receptor binding domain protein

### Determining the reference values (cutoff) for the insect expressed S2 extracellular domain of the SARS-CoV-2 spike protein for the CoV seroassay

**Fig. 3:**
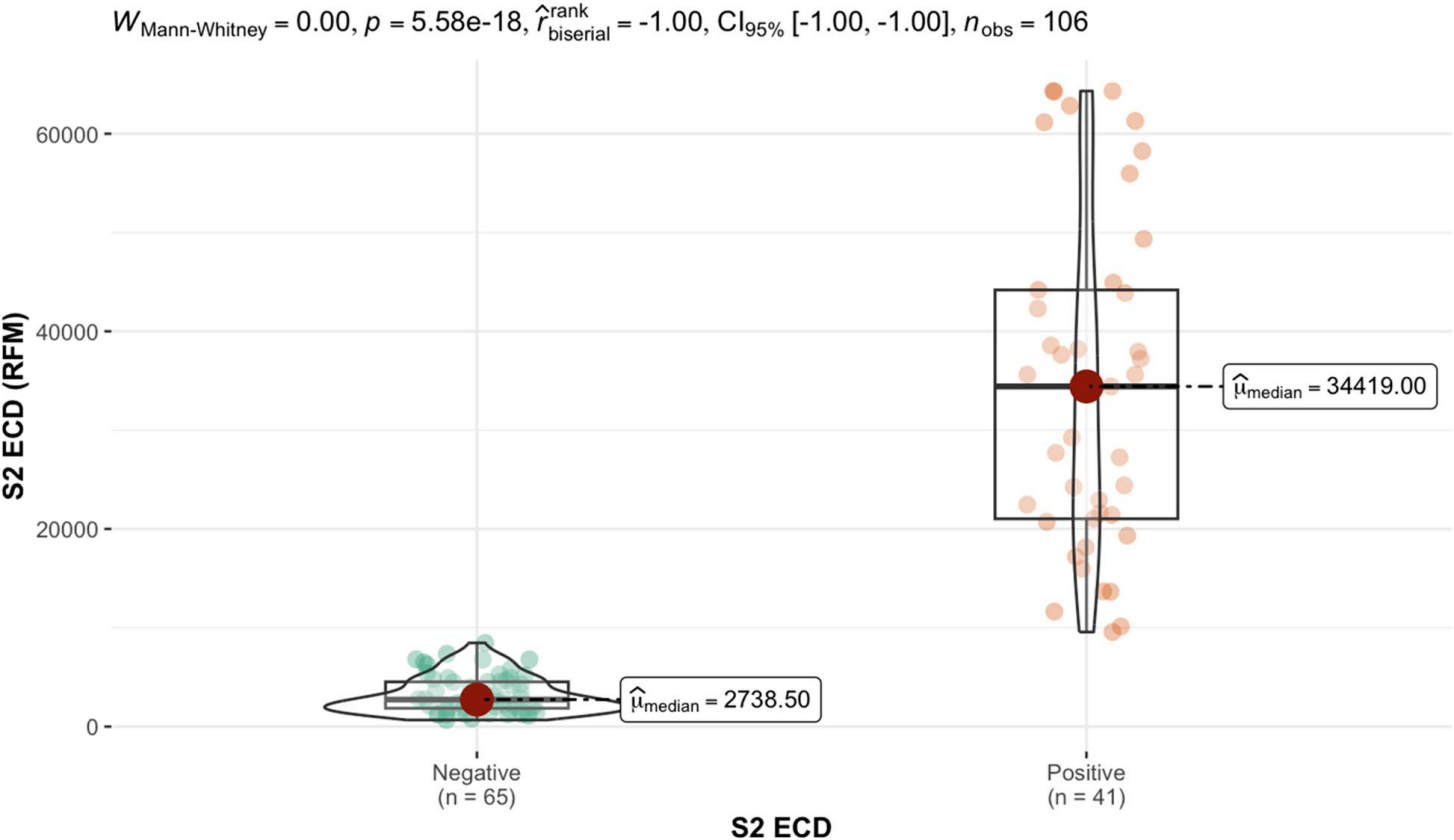
Mann–Whitney test of the distribution of relative fluorescent means (RFM) from S2 extracellular domain (ECD). Significant statistical difference (*p*-value < 0.000) between the positive (34,419) and negative (2738.5) RFM median based on reference value (cutoffs)

The CoV seroassay and InDevR instrument were used to test 106 individual serums. The relative fluorescent mean (RFM) results for the S2 ECD protein and nucleocapsid assay results are shown in Table 6. Out of the 106 individuals tested, 37.7% generated a positive response and 62.3% were considered a negative response based on nucleocapsid results listed in Table 6. The nucleocapsid assay identified individuals that were not previously infected with SARS-CoV-2. Twenty random individuals with a negative response in the nucleocapsid assay were considered negative controls. The designated negative control RFM values were used to calculate the upper tail of the Student t-distribution method for five distinct confident intervals (95.0%, 97.5%, 99.0%, 99.5%, 99.9%) for the S2 extracellular domain protein in Table 7. The 9096 RFM at 95% confidence interval was determined as the reference value (cutoff). Out of the 106 individuals tested, 38.7% generated a positive response and 61.3% were considered a negative response based on reference values cutoff results listed in Table 7. Relative fluorescent mean readings above 9096 are classified as positive, while readings at or below the relative value are negative. The median from positive S2 ECD individuals is 34,419 RFM and the median for negative individuals is 2738.5 RFM. Applying the Mann–Whitney test to the RFM data in Table 6 determined there is a significant statistical difference between (*p*-value < 0.000) the distribution of RFM medians between positive and negative individuals is depicted (Fig. 3.)

**Table 6:**
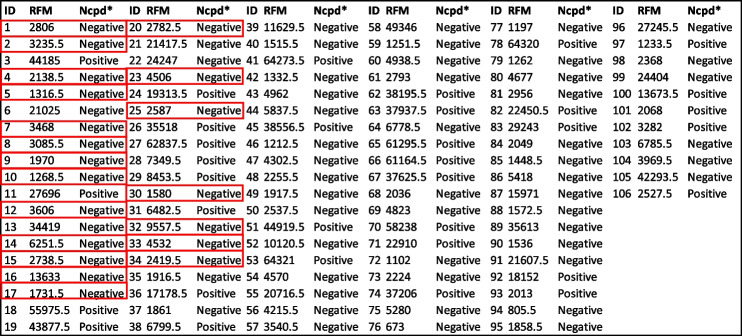
S2 extracellular domain (ECD) Relative fluorescent mean (RFM) for the CoV2 Seroassay and nucleocapsid ELISA results from 106 individual serums

^*^Nucleocapsid assay results

negative controls

**Table 7:** The quantitative determination of the S2 extracellular domain protein reference value (cutoff)

Standard deviation multipliers (*f*) for calculation reference value					
Number of Controls	95.0%	97.5%	99.0%	99.5%	99.9%
2	1554.96	1820.69	2615.25	3938.66	14,524.35
3	1953.82	2221.58	2737.09	3310.38	5712.20
4	2051.74	2255.23	2588.60	2907.72	3981.19
5	2255.45	2461.71	2772.25	3046.52	3868.58
6	2426.16	2635.32	2934.39	3185.59	3886.36
7	2661.24	2891.00	3208.35	3465.95	4150.21
8	2857.73	3101.19	3429.23	3688.99	4354.98
9	3030.02	3283.99	3619.87	3880.92	4532.48
10	3148.49	3404.83	3738.99	3994.95	4620.49
11	3231.315	3485.803	3813.73	4061.989	4658.558
12	3368.968	3632.203	3968.211	4220.157	4817.255
13	3507.589	3780.371	4125.839	4382.81	4984.813
14	3670.448	3957.247	4318.061	4584.631	5203.03
15	3825.285	4124.955	4499.825	4775.17	5408.576
16	4192.506	4541.115	4975.063	5292.195	6016.427
17	4465.076	4844.828	5315.513	5657.976	6435.076
18	5165.035	5649.154	6246.924	6680.158	7657.705
19	6740.868	7481.283	8392.441	9050.517	10,527.97
20	9095.523^a^	10,218.23	11,595.68	12,587.44	14,804.1

^a^Relative fluorescents mean cutoff for the S2 extracellular domain protein

### Relationship of antibodies recognizing FLS, RBD, and S2 ECD in the SARS-CoV-2 vaccine

**Fig. 4:**
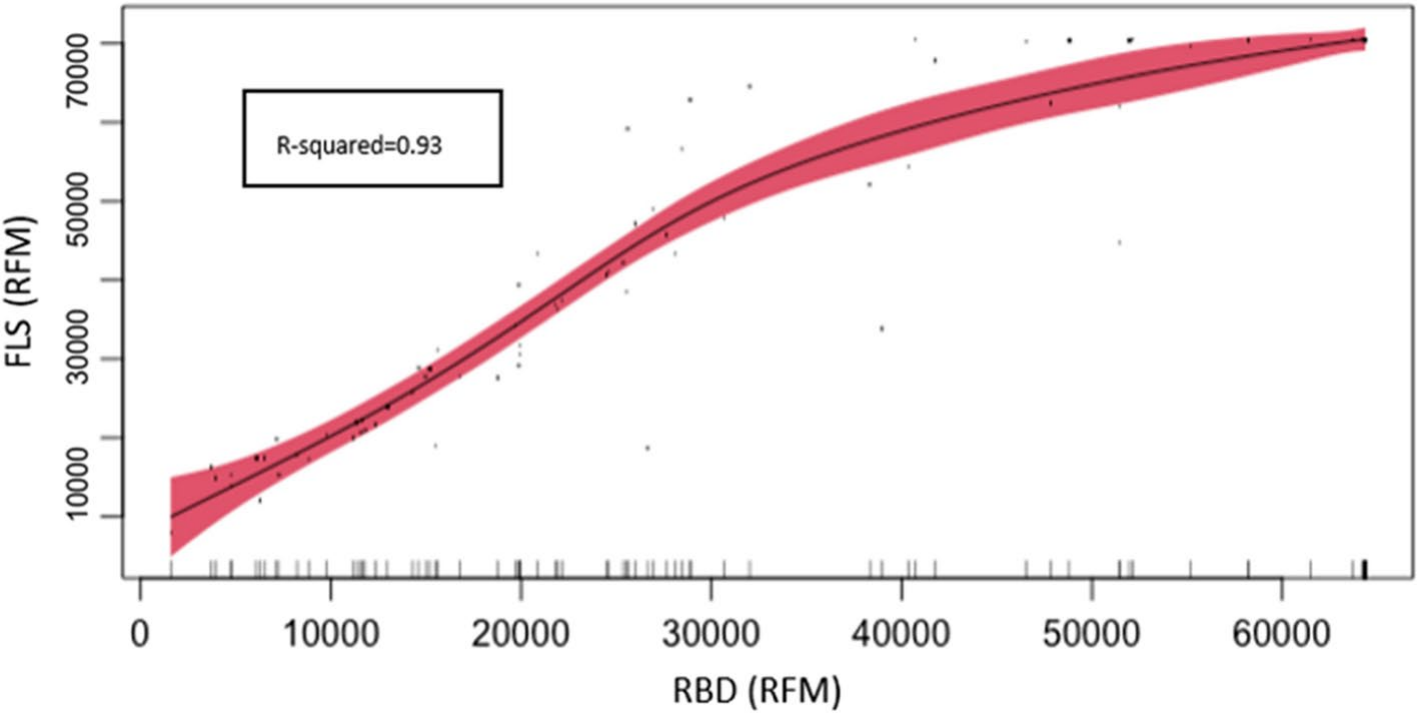
Generalized non-parametric regression based on CoV Seroassay from FLS and RBD antigens. The graph displays full length spike antigen (FLS) and receptor binding domain antigen (RBD) relative fluorescent mean (RFM) values for 106 individual serums tested using the CoV seroassay. The graph shows direct and statistically significant (*p*-value < 0.000) association between FLS and RBD

It has been shown that vaccination generates antibodies against the distinct domains of the spike protein. Although both FLS and RBD antigens are expressed in mammalian systems and the S2 ECD antigen is expressed in baculovirus both expression systems have the capability to preserve post-translational modifications. It has been previously demonstrated that RBD is the most immunodominant domain in the spike protein [10]. To determine how the FLS reference value (cutoff) is impacted from antibodies recognizing regions outside of the RBD, we performed generalized non-parametric regression between FLS and RBD using RFM values from the CoV Seroassay (Fig. 4.). This analysis supports the understanding that the RBD is an immunodominant domain in the FLS protein. RFM results from RBD can predict FLS antigen signal due to the statistically significant (*p*-value < 0.000) association between FLS and RBD, with a R-squared value of 93.2%. RBD RFM values can explain 93.2% of the FLS values.

### Utilization of the reference value (cutoff) for FLS, RBD and S2 ECD antigens leads to distinguishing vaccine and natural infection immune response

The different combinations of positive or negative results among the first three antigens are listed in (Table 8). This table provides an list of outcomes and interpretations of the results from the CoV seroassay. In outcome 1, the immune response suggests that the individual had an IgG immune response due to vaccination (RBD +) and natural infection (S2 ECD +). In outcome 2, the immune response suggests that an individual had an IgG immune response due to vaccination (RBD +) and not due to natural infection (S2 ECD -). In outcome 3, the immune response suggests that an individual had an IgG immune response due to natural infection (S2 ECD +) and not to vaccination (RBD -). In outcome 4, the immune response suggested that the individual did not have an IgG immune response due to natural infection (S2 ECD-) or vaccination (RBD-).

**Table 8:** Immune response outcomes from vaccinated individuals

Antigens	Full length Spike FLS	RBD	S2 ECD	Interpretation
Outcome 1	** + **	** + **	** + **	Natural infection and Vaccine positive immune response
Outcome 2	** + **	** + **	**-**	Vaccine positive immune response
Outcome 3	** + **	**-**	** + **	Natural infection positive immune response
Outcome 4	**-**	**-**	**-**	Natural infection and vaccine negative immune response

## Discussion

This study provides a statistical approach to estimate reference values for serological studies.

Serological studies commonly use many samples to estimate and validate reference values for assays, typically presented as a limited dilution series of two to three standard deviations from the mean negative control reading. This study has presented statistically established reference values determined by the upper tail of the Student *t*-distribution method that characterizes vaccine and natural infection immune responses utilizing the CoV-2 seroassay. This method allowed us to determine reference values with a limited number of negative controls and with an adjustable confidence level that researchers can utilize according to their needs.

In our study we attempted to distinguish vaccine and natural SARS-CoV-2 immune responses using off the shelf consumer available assays. It is known that vaccination generates antibodies against all distinct domains of the spike protein and RBD is the most immunodominant domain in the full-length spike protein. We also observed this RBD immunodominance based on our generalized non-parametric regression analysis on FLS which supports the rationale that antibodies recognizing regions outside of the RBD will not significantly impact the FLS reference value (cutoff) estimation. Our method also characterized the antibody response against all key domains included in the vaccine from individuals who were not previously infected but vaccinated determined the background signal required to calculate the upper student-t distribution for the determination of the reference value cutoffs for the S2 ECD antigen.

The current state of SARS-CoV-2 among population regarding immunization strategy including vaccination rate, vaccine efficacy and effectiveness necessitates continue research and use of serological testing to understand the impact of SARS-COV-2 variants in the level of protection, viral inhibition and longevity of the immune response generated from vaccine and natural infection. The translation of this knowledge is crucial to the public health immunization programs to increase public awareness about vaccine protection and health outcomes as well as assess the risk associated to low immunization rate and its impact in the healthcare system.

In this regard, future assays are needed to include variant specific antigens and nucleocapsid. Based on our study, these key antigens are needed to evaluate and estimates statistically significant reference values (cutoffs) to characterize vaccine and natural infection immune response as well as neutralizing capacity and viral inhibition against to the new strain circulating.

## Supplementary Information

Supplementary Material 1.

Supplementary Material 2.

## Data Availability

Data is provided within the manuscript or supplementary information.
