# Supplementary material for: A statistically established reference value determined for the Vaxarray Coronavirus (CoV) seroassay to characterize vaccination and natural infection
Source: BMC Infect Dis. 2024 Nov 15;24:1308. doi: 10.1186/s12879-024-10117-5 (PMC11566399; doi:10.1186/s12879-024-10117-5)
Supplement: Supplementary file 1 — Supplementary Material 1. [file 12879_2024_10117_MOESM1_ESM.docx]

**##Cutoff Calculations Reference:Frey.A.1998 "A Statistically defined endpoint titer determination method for #immunological Methods"

#The R code for calculating the upper Student t-distribution method.
## Reference Value Calculations
### Frey Method

library(readxl) ### excel reading library
library(dplyr)  ### data manipulation library among other functions.
 
#FLS-Reference Value (95% confidence level)
m <- read_excel("/Users/Write Computer Username/Desktop/Data_publication.xlsx",sheet = "FLS")
View(m)
OD1 <- m
OD1 <- select(OD1,FLS)
View(OD1)
m=mean(OD1$FLS)
s=sd(OD1$FLS)
n=length(OD1$FLS)
df=n-1
t=qt(0.05, df=df, lower.tail = FALSE)  #95% confidence level
f=t*(sqrt(1+(1/n)))
Reference_value1 =m+(f*s)
Reference_value1

#RBD-Reference Value (95% confidence level)
m1 <- read_excel("/Users/Write Computer Username/Desktop/Data_publication.xlsx",sheet = "RBD")
View(m1)
OD2 <- m1
OD2 <- select(OD2,RBD)
View(OD2)
m=mean(OD2$RBD)
s=sd(OD2$RBD)
n=length(OD2$RBD)
df=n-1
t=qt(0.05, df=df, lower.tail = FALSE)  #95% confidence level
f=t*(sqrt(1+(1/n)))
Reference_value2 =m+(f*s)
Reference_value2
 
#S2 ECD-Reference Value 95% confidence level
m1 <- read_excel("/Users/Write Computer Username/Desktop/Data_publication.xlsx",sheet = "S2ECD")
View(m1)
OD3 <- m1
OD3 <- select(OD3,S2ECD)
View(OD3)
m=mean(OD3$S2ECD)
s=sd(OD3$S2ECD)
n=length(OD3$S2ECD)
df=n-1
t=qt(0.05, df=df, lower.tail = FALSE)  #95% confidence level
f=t*(sqrt(1+(1/n)))
Reference_value3 =m+(f*s)
Reference_value3**

Supplemental Figure 1.

The R code for calculating the upper tail of the Student *t*-distribution method.
